# Reversible Nuclear-Lipid-Droplet Morphology Induced by Oleic Acid: A Link to Cellular-Lipid Metabolism

**DOI:** 10.1371/journal.pone.0170608

**Published:** 2017-01-26

**Authors:** Lucía C. Lagrutta, Sandra Montero-Villegas, Juan P. Layerenza, Martín S. Sisti, Margarita M. García de Bravo, Ana Ves-Losada

**Affiliations:** 1 Instituto de Investigaciones Bioquímicas de La Plata “Profesor Doctor Rodolfo R. Brenner” (INIBIOLP), La Plata, Buenos Aires, Argentina; 2 Cátedra de Biología, Facultad de Ciencias Médicas, Universidad Nacional de La Plata; La Plata, Buenos Aires, Argentina; 3 Departamento de Ciencias Biológicas, Facultad de Ciencias Exactas, Universidad Nacional de La Plata, La Plata, Buenos Aires, Argentina; University of Basque Country, SPAIN

## Abstract

Neutral lipids—involved in many cellular processes—are stored as lipid droplets (LD), those mainly cytosolic (cLD) along with a small nuclear population (nLD). nLD could be involved in nuclear-lipid homeostasis serving as an endonuclear buffering system that would provide or incorporate lipids and proteins involved in signalling pathways as transcription factors and as enzymes of lipid metabolism and nuclear processes. Our aim was to determine if nLD constituted a dynamic domain. Oleic-acid (OA) added to rat hepatocytes or HepG2 cells in culture produced cellular-phenotypic LD modifications: increases in TAG, CE, C, and PL content and in cLD and nLD numbers and sizes. LD increments were reversed on exclusion of OA and were prevented by inhibition of acyl-CoA synthetase (with Triacsin C) and thus lipid biosynthesis. Under all conditions, nLD corresponded to a small population (2–10%) of total cellular LD. The anabolism triggered by OA, involving morphologic and size changes within the cLD and nLD populations, was reversed by a net balance of catabolism, upon eliminating OA. These catabolic processes included lipolysis and the mobilization of hydrolyzed FA from the LD to cytosolic-oxidation sites. These results would imply that nLD are actively involved in nuclear processes that include lipids. In conclusion, nLD are a dynamic nuclear domain since they are modified by OA through a reversible mechanism in combination with cLD; this process involves acyl-CoA-synthetase activity; ongoing TAG, CE, and PL biosynthesis. Thus, liver nLD and cLD are both dynamic cellular organelles.

## Introduction

In Eukaryotic cells the nucleus is a highly dynamic organelle where the processes of replication, transcription, RNA splicing, and ribosome assembly—among other fundamental cellular functions—take place.

Within the nucleus, the nuclear matrix is circumscribed by the nuclear envelope and contains chromatin and two different types of domains, with the two having a specific supramolecular structure: the nuclear bodies—such as the nucleolus, the Cajal body, speckles, premyelocytic leukemia, those being mainly composed by proteins and RNA [1]—and the nuclear lipid droplets (nLD) that we have previously described and characterized under normal and nonpathologic conditions [2]. nLD have different morphologic characteristics and functional properties compared to nuclear bodies, since under nonpathologic conditions the nLD would be the only nuclear domain surrounded by a lipoprotein monolayer and mainly composed of lipids [2].

Under normal and nonpathologic conditions, nLD are a very small nuclear domain consisting of a few small lipid droplets (LD) randomly distributed within the nucleus that can be visualized only after staining. nLD are smaller and have a different proportion of the same neutral-lipid classes than the cytoplasmic lipid droplets (cLD)—*i*. *e*., polar lipids (PL) triacylglycerides (TAG), cholesterol esters (CE) and cholesterol (C) [2]. nLD have also been observed in the nuclei of mice fed a high-fat diet [3] as well as under different pathologic conditions [4] such as in mouse embryos bearing a lethal mutation [5], in a liver patient with chronic hepatitis-C virus [6], and in carcinogen-fed rats [7] and mice [8].

nLD could be involved in nuclear-lipid homeostasis and serve as an endonuclear buffering system that would provide or incorporate lipids and proteins involved in signaling pathways, ligands for transcription factors, and enzymes catalyzing lipid metabolism and nuclear processes. Thus, within the nucleus, the lipids would have two main locations: the nuclear envelope—composed mainly of glycerophospholipids, sphingolipids, and C—and the nLD (containing principally TAG, CE, C, and PL). Both of these domains would constitute alternative lipid sources with different chemical compositions, physical properties, potential functions, and possible regulatory characteristics.

The aim of the present work was to determine if nLD constituted a dynamic domain. Our hypothesis was that the nLD were just such a domain within the nucleus that accordingly responded to external cellular stimuli, such as the LD in the cytosol.

We demonstrate here that nLD are a dynamic nuclear domain since they are modified by oleic acid (OA) through a reversible mechanism in combination with the cLD. This process involves acyl-CoA-synthetase activity; ongoing TAG, CE, and PL biosynthesis; LD-morphologic changes; and LD population size.

## Materials and Methods

### Materials

Collagenase IV, fatty acid-free bovine-serum albumin (BSA), OA sodium salt (≥99% purity), Triacsin C (long-chain acyl-CoA-synthetase inhibitor), and 4',6-diamidino-2-phenylindole (DAPI) were obtained from Sigma-Aldrich (St. Louis, MO, USA); BODIPY 493/503 from Invitrogen (Buenos Aires, Argentina); silica-gel-G–precoated 20-x-20-cm thin-layer-chromatography plates from Merck (Buenos Aires, Argentina); and lipid standards from Nu-Chek Prep, Inc. (Elysian, MN, USA). All chemicals and solvents were of analytical and HPLC grade, respectively.

### Cell culture

#### HepG2 cell culture

HepG2 human-hepatoma cells from the American Type Culture Collection (ATCC HB-8065) were maintained and grown in a humidified incubator at 5% (v/v) CO2/air and 37°C in 75-cm^2^ flasks. Cells were plated on coverlips in 6-well plates at a density of 2 x 10^5^ cells *per* well and cultured in filter–sterilized Eagle's Minimum Essential Medium containing 2 mM L-glutamine, 2.2 g.l^−1^ sodium bicarbonate, 0.1 mM nonessential amino acids, and 1.0 mM sodium pyruvate (Gibco, Invitrogen corporation) and supplemented with 10% (v/v) fetal-bovine serum (Natocor, Córdoba, Argentina) plus 0.1 g.l^−1^ streptomycin.

#### Primary culture of hepatocytes

The isolation of rat hepatocytes was performed on 200- to 250-g 60- to 80-day-old male Wistar rats. Rats were housed in rooms with 12:12 h light-dark diurnal cycle (midnight being the midpoint of the dark period), and the experiments were performed following the Animal Welfare Guidelines of NIH (INIBIOLP’s Animal Welfare Assurance No A5647-01). The corresponding protocol was approved by our Institutional Animal Welfare Committee, (Comité Institucional para el Cuidado y Uso de Animals de Laboratorio: CICUAL) protocol # P05-02-2015. The rats were maintained on a commercial standard pellet diet (ACAI mouse and rat chow; San Nicolás, Buenos Aires, Argentina) plus tap water *ad libitum*. The diet contained (by weight) 20% proteins and 4% total lipids with a fatty-acid (FA) composition of 15.6% 16:0; 1.1% 16:1; 6.3% 18:0; 26.9% 18:1 (n-9); 1.6% 18:1 (n- 7); 42.7% 18:2 (n- 6); 5.7% 18:3 (n- 3); and trace amounts of 14:0, 20:4 (n-6), and 22:6 (n- 3).

The isolation of hepatic cells from the livers of donor animals was performed via the two-step enzymatic method described by Seglen [9] with slight modifications; for this purpose, a dual-channel system (initially open and then closed) was used to effect the initial washing and final organ digestion. Rats were deeply anesthetized by a subcutaneous injection of ketamine (Ketamina 50, Holliday, 75 mg.kg^−1^ weight) and diazepam (Diazepam, Lamar, 5 mg.kg^−1^ weight) and then the abdomen was surgically opened. The procedure basically consisted in an organ perfusion *in situ* at 37°C with a washing solution lacking Ca^++^ and Mg^++^ and supplemented with EGTA to chelate those divalent ions and weaken the intercellular junctions, followed by the perfusion of an enzymatic solution of 0.025% (w/v) type-IV collagenase (at 37°C) to digest the intercellular matrix. The liver was then removed from the animal. The explanted liver was transferred to a sterile Petri dish in a laminar-flow hood, where the organ was teased apart mechanically. The resulting cell suspension—in Hanks's balanced salt solution (0.14 g.l^−1^ CaCl_2_, 0.01 g.l^−1^ MgSO_4_, 0.4 g.l^−1^ KCl, 0.06 g.l^−1^ KH_2_PO_4_, 8 g.l^−1^ NaCl, 0.05 g.l^−1^ Na_2_HPO_4_, 1.0 g.l^−1^ D-glucose)—was passed through a sieve into a 50-ml tube. After addition of approximately 20 ml of the Hanks solution to the filtered cell suspension, the latter was centrifuged at 50 x *g* for 3 min. Trypan-blue dye exclusion was used to ascertain the viability of the isolated cells [10].

Hepatocytes were plated at a density of 2.5 x 10^5^ cells *per* well in 6-well plates and cultured in Dulbecco's modified Eagle's medium with high glucose (Gibco, Grand Island, NY, USA), supplemented with 10% fetal-bovine serum, plus 100 U.ml^−1^ penicillin and 0.1 g.l^−1^ streptomycin. The medium was renewed after 6 h and thereafter every 24 h.

### Cell-culture treatments

**Fig 1:**
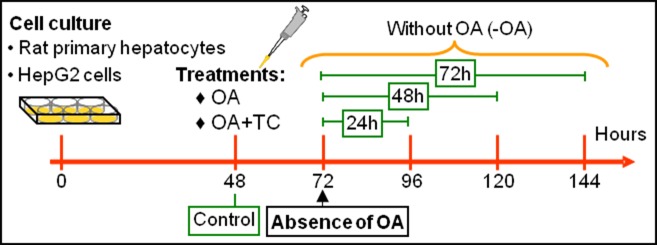
Experimental protocol for the different treatments used with rat primary hepatocytes and HepG2 cells. Cells treated with the respective vehicle of OA or Triacsin C, ethanol+BSA or methanol, constituted the controls for the different experimental groups.

HepG2 cells and hepatocytes were cultured in the respective media described above supplemented with 10% fetal-bovine serum as reported previously [2]. Confluent cells were treated with either OA (100 and 400 μM) alone or OA (400 μM) plus the lipid-biosynthesis inhibitor Triacsin C (TC; at 1, 2.5, or 5 μM) for 24 h. In some instances, the cells were cultured with OA (400 μM) for 24 h then incubated for a further 24, 48, or 72 h in fresh medium without OA (Fig 1). In all treatments fresh medium was provided every 24 h. The OA dissolved in ethanol was added to the medium complexed with BSA at a molar ratio of 6:1 OA:BSA. The TC was added dissolved in methanol. A total medium volume of 2 ml per well was used in all experiments.

### Cell viability

Viability was determined by trypan-blue–dye-exclusion cell counts in a Neubauer chamber [10] and by the MTT assay [10] as follows:

#### MTT assay

The MTT assay was performed as described previously [10]. hepatocytes and HepG2 cells were seeded in 24-well plates (2 x 10^4^ cells per well), grown under standard conditions for 48 h, and then treated with OA with or without TC for 24 h as described in *Cell-culture treatments* above. The mitochondrial metabolic activity in the viable cells was determined by the conversion of MTT (thiazolyl blue tetrazolium bromide; M2128, Sigma) to formazan. That reaction product was dissolved in 0.04 M HCl in isopropanol and the absorbance measured at 560 nm with an Elisa reader (Beckman Coulter DTX 880 Multimode Detector).

#### Cell counting

Cells were seeded and treated as described for the MTT method. After treatment, the cells were harvested by trypsinization and the cell suspensions mixed (1/1, v/v) with 0.4% (w/v) trypan-blue solution.

### Lipid analysis

Lipids, extracted by the procedure of Folch et al. [11], were recovered from the chloroform layer and separated into different classes by thin-layer chromatography on plates precoated with silica gel G. For the separation of the lipids, 80:20:1 (v/v/v) hexane/diethylether/acetic acid was used as the mobile phase [2]. After visualization of the lipid species by exposure to iodine vapor, the PL, diacylglycerides (DAG), C, FA, TAG, and CE were located by comparison to the corresponding positions of known standards having the respective retention factors of 0.06, 0.13, 0.18, 0.27, 0.60, and 0.96.

The PL (glycerophospholipids + sphingolipids) were scraped off the plate for quantification by the spectrophotometric determination of phosphorus. To separate and quantify the C and CE, the sample was developed on thin-layer chromatography plates along with different concentrations of the pure standards, the lipid spots revealed after spraying with acidic ferric-chloride solution, and the staining densities quantified through the use of ImageJ software (National Institutes of Health, Bethesda, MD, USA).

The TAG scraped off the plates were quantified by means of an assay-kit procedure based on the release of glycerol through a lipase-catalyzed hydrolysis (TAG Color, Wiener Lab., Rosario, Santa Fe, Argentina) as previously described [2].

### Bright-field microscopy

HepG2 cells and rat-liver hepatocytes grown on coverslips were kept on ice and fixed by an overnight incubation with 4% (w/v) paraformaldehyde in PBS buffer (8 g.l^−1^ NaCl, 0.2 g.l^−1^ KCl, 0.2 g.l^−1^ KH_2_PO_4_, 3.14 g.l^−1^ NaH_2_PO_4_.12H_2_O) or Hanks’s solution at room temperature, respectively. Samples were examined by bright-field microscopy under an Olympus BX51 epifluorescence microscope (Shinjuku, Tokyo, Japan) through the use of the Image-Pro plus version 5.1 software (Media Cybernetics Inc, Bethesda, MD, USA).

### Confocal laser-scanning microscopy

Coverlips with HepG2 cells or rat-liver hepatocytes were fixed as described for bright-field microscopy above. Samples were washed 3 times with the respective buffer leaving each wash on the cells successively for 10, 10, and 5 min. Permeabilization was effected by incubation with 0.08% (v/v) Triton X-100 in the same buffer for 20 min. Samples were then washed 4 times with that buffer with similar sequential incubations of 10, 10, 10, and 5 min per wash. The samples were incubated with DAPI (final concentration, 1 μg.ml^−1^) and BODIPY 493/503 (final concentration, 1 μg.ml^−1^) for 1 h. After three 10-min washes with PBS buffer or Hanks’s solution, the samples were mounted and observed. Images of fixed HepG2 cells and rat-liver hepatocytes were examined with an Olympus (Shinjuku, Tokyo, Japan) confocal laser-scanning microscope (Fluoview FV1000, software version 1.7.3.0) mounted on an Olympus BX61 upright frame microscope, equipped with a 405-nm, a 473-nm, and a 559-nm diode laser and containing an UPLSAPO 40X (0.95 numerical aperture) objective. Ten representative fields containing several cells were selected. To visualize nuclear-domains, Z-planes of 0.20- or 0.68-μm thickness were acquired. Z-plane cross sections across the central position of a nucleus were obtained and the different planes digitally reconstructed.

### Image analysis and quantification of lipid droplets

To quantify the abundance (numbers) of nLD and cLD and estimate their sizes, images of cells stained with BODIPY 493/503 and DAPI were analyzed by means of the Image-Pro plus version 5.1 and ImageJ (National Institutes of Health, Bethesda, MD, USA) software. The nLD and cLD areas were manually drawn and used to determine each LD diameter and LD number in the nucleus (for the nLD) and cytosol (for the cLD). In this manner, the maximum, minimum, and median nLD and cLD diameters were calculated as described in the following section; and from those last dimensions, the total volume for the nLD and cLD populations present after each cell culture treatment was obtained as the product between the median LD volume and the mean LD number for each respective compartment.

For 3-dimensional reconstructions we used ImageJ and Amira ResolveRT 4.0 (Visaje Imaging Inc., San Diego, CA, USA) software.

### Data analysis

The results are the mean values of at least three or five independent determinations ± SD.

In order to find differences among the parametric data, the ANOVA test with *post-hoc* comparisons of the means (Tukey's honestly-significant-difference test [HSD]) and the Student's *t* test were employed.

At least 10 fields (of the fluorescence-microscopical images) with a variable number of cells in each were analyzed to determine the LD morphologic parameters and quantify their total number in the nucleus (the nLD) and cytosol (the cLD). With the latter calculation, in order to determine the number of cLD per cell, we counted the total number of cLD per field and then divided by the number of nuclei present.

The total volume of the nLD and cLD per cell was calculated under the assumption that the LD were spheres by multiplying the volume of an ideal LD (its diameter being the median value for the population) by the total number of LD (mean) within the population in the nucleus (nLD total volume) and cytosol (cLD total volume). The maximum diameter (d_max_) of each population (*i*. *e*., the nLD and cLD) was also recorded. The minimum diameter could not be evaluated because that dimension corresponded to the resolution limit of the microscope used and thus was always the same for both populations.

To determine parametric or nonparametric data, the Kolmogorov-Smirnov test was used. If the data were nonparametric, the Kruskal–Wallis test with *post-hoc* comparisons of the medians was performed (*i*. *e*., the Nemenyi test). To compare proportions in the analysis of the diameter distribution, multiple-significance tests with the Bonferroni correction were used.

Statistical significance was set at P <0.05; calculations were carried out with the SPSS 15.0 software for Windows (IBM Corp. (2006), Somers, NY, USA).

## Results

### Cellular-LD morphologic characterization: nLD and cLD

The first objective was to characterize the cellular LD populations morphologically—*i*. *e*., the cLD and nLD—in a liver model under conditions that are, at once, physiologic, nonpathologic, and unstimulated as well as to define measurable parameters for use in comparative studies of these LD populations. Most studies on LD are made in cells treated with OA or stimulated in some way [2–8]; but although those physiologic states have constituted useful methodologic tools, under such conditions the phenotype of the LD populations, at the very least, become modified. In general, cLD populations have not been analyzed under control conditions involving basal physiology [2–8]—and nLD populations even less so.

**Fig 2:**
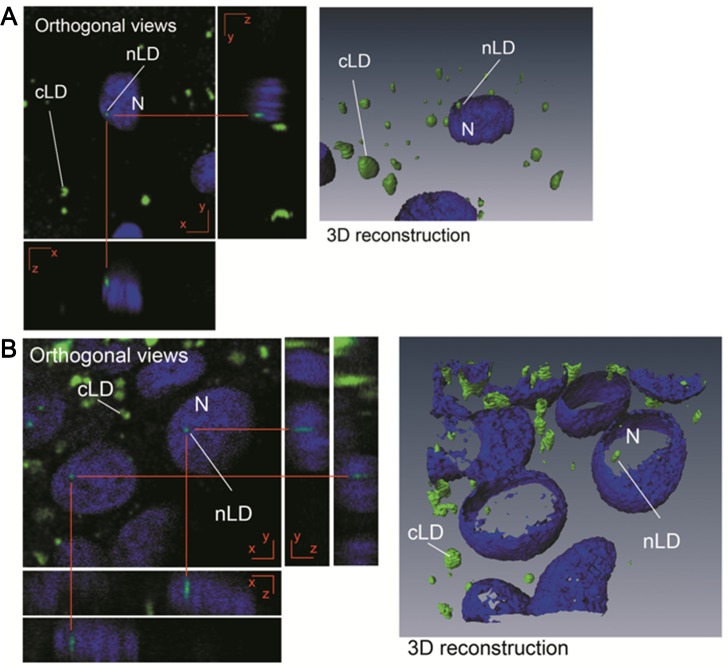
Analysis of primary rat hepatocytes and HepG2 cells by microscopy. (**A**) Rat primary hepatocytes; (**B**) HepG2 cells, both cultured under control conditions. Nuclei and LD (nLD and cLD) were stained with DAPI (blue) and BODIPY 493/503 (green), respectively, and observed by confocal laser-scanning microscopy. Z-plane cross sections through the central position of a nucleus were obtained and the different planes reconstructed to give XZ and YZ orthogonal projections. Three-dimensional reconstructions were performed and the nLD visualized through isosurface rendering by BODIPY (green) and outline rendering by DAPI (blue). The photographs correspond to representative observations. N, nucleus.

As a first step, primary hepatocytes and HepG2 cells cultured under control physiologic conditions were analyzed by bright-field microscopy (S1 Fig) and confocal laser-scanning microscopy (Fig 2), for which latter visualization the nuclei were stained with DAPI and the LDs with BODIPY 493/503.

The two cell types in culture exhibited morphologic characteristics of hepatocytes—namely, a polygonal shape and growth in an epithelial monolayer (S1 Fig). The nuclear location of the nLD was confirmed by confocal microscopy (Fig 2), but in both the hepatocytes and the HepG2 cells, the LD were located in the cytosol (cLD) as well as in the nucleus (nLD). The nLD were randomly distributed and were fewer in number than the cLD, in agreement with previous results described for rat-liver squashes, isolated rat-liver nuclei, and HepG2 cells [2]. The primary rat hepatocytes remained viable and with all their normal morphologic characteristics for at least 6 days in culture.

#### Hepatocytes

**Fig 3:**
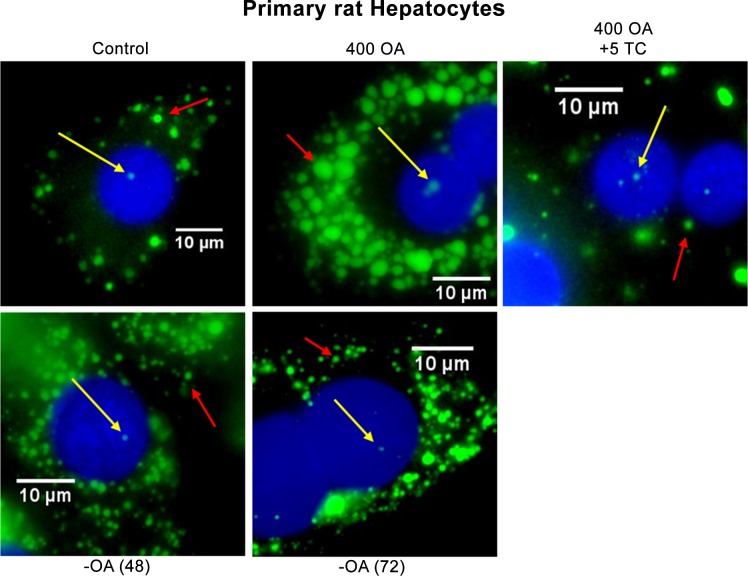
Analysis of cLD and nLD populations from rat primary hepatocytes by fluorescence microscopy. Nuclei (N) and lipid droplets (nLD and cLD) were stained with DAPI (blue) and BODIPY 493/503 (green), respectively. The red and yellow arrows indicate the cLD and nLD, respectively. Control, cells cultured under control conditions for 24 h; 400 OA, cells treated with 400 μM OA for 24 h; 400 OA + 5TC: cells treated with 400 μM OA plus 5 μM TC for 24 h;–OA (48) and–OA (72), cells treated with 400 μM OA for 24h and then incubated in the absence of OA for 48 or 72 h, respectively. The photographs correspond to representative observations.

**Fig 4:**
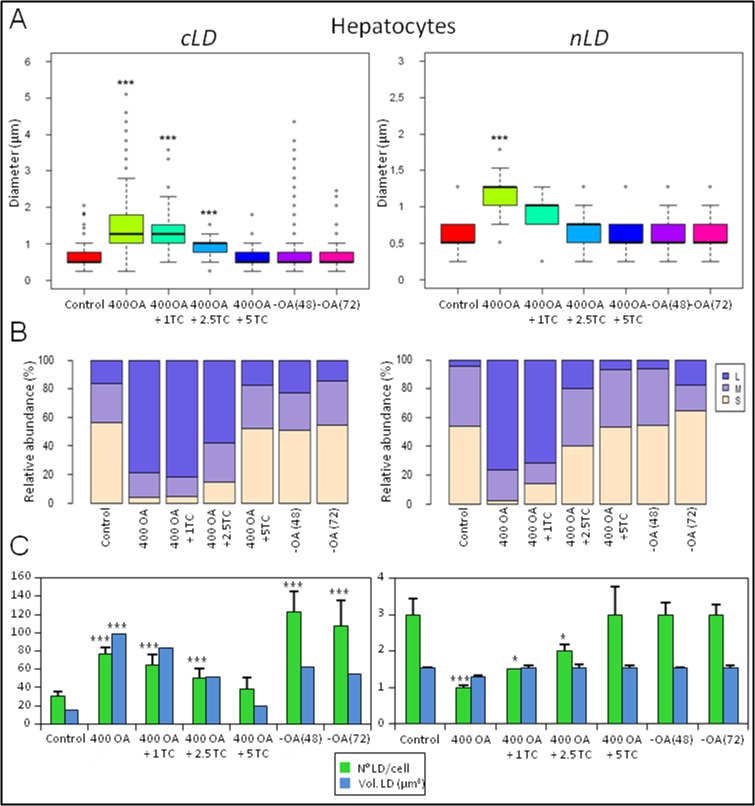
Analysis of the cLD and nLD morphologic parameters of rat hepatocytes in primary culture. The morphologic parameters of hepatocyte-LD populations (cLD and nLD) were measured in fluorescence-microscopy images (equivalent those of to Fig 3) through the use of Image-Pro Plus software as described in the Materials and Methods section. Experimental groups, Control: cells cultured in medium plus ethanol (solvent in which OA was dissolved), BSA (OA vehicle) and methanol (TC vehicle) for 24 h; OA 400: cells treated with 400 μM OA for 24 h; OA 400 + 1TC, 2.5TC, or 5TC: cells treated with 400 μM OA plus 1, 2.5, or 5 μM TC, respectively for 24 h;–OA (48) and–OA (72): cells treated with 400 μM OA for 24 h and then incubated in the absence of OA for 48 or 72 h, respectively. (**A**) LD sizes (diameters). The diameter values measured for cLD and nLD under each experimental condition are represented by box plots; where the minimum, median (indicated as a black line across the bar), and maximum values are displayed and the three quartiles of the data (Q2 = median value). The maximum diameter (d_max_) corresponds to the maximum diameter measured in the cLD or nLD population. The statistical significance of differences in the data was analyzed by the Kruskal-Wallis test with *post-hoc* comparisons of the medians (the Nemenyi test). Each treatment was compared with the corresponding control condition: *p <0.05, **p <0.01, ***p <0.001. In the figure the LD diameter is plotted on the *ordinate* for each of the experimental groups indicated on the *abscissa*. Note the difference in the size scales on the left (cLD) and right (nLD) *ordinates*. (**B**) Size distribution of LD populations. The sizes of the cLD and nLD populations were evaluated by defining three categories according to the quartiles of the diameter data, Q1 (0.51 μm) and Q3 (0.77 μm) as follows: small, (beige bar area), ≤0.51; medium, (light-violet bar area), between 0.51 and 0.77 (>0.51 and ≤0.77); large, (dark-violet bar area), >0.77. To compare the LD size distribution, the data were analyzed by multiple significance tests with the Bonferroni correction (see S1 and S2 Tables). Each experimental treatment was compared with the corresponding control condition for the same LD-size category (small, medium, or large; *p<0.05, **p<0.01, ***p<0.001). In the figure, the relative abundance as a percent of each size class is plotted on the *ordinate* for each of the experimental groups indicated on the *abscissa*. (**C**) Total numbers and volumes of the LD populations. The total volume of each LD population (μm3) was calculated under the assumption that the LD were spheres by multiplying the volume of an ideal LD (*i*. *e*., one having the median diameter of the population) by the mean number present in the cytoplasm (cLD) and nucleus (nLD). The statistical significance of differences in the number and volume was analyzed by the Student's *t* test and the Kruskal-Wallis test with *post-hoc* comparisons of the medians (the Nemenyi test), respectively. Each treatment was compared with the corresponding control condition: *p <0.05, **p <0.01, ***p <0.001. In the figure, the number and volume (μm3) of LD are plotted on the *ordinate* for each of the experimental groups indicated on the *abscissa*.

The rat-liver hepatocyte in primary culture under control physiologic conditions has a mean of 31 cLD in the cytoplasm and 3 nLD in the nucleus (Fig 3 and Fig 4). Both LD populations (the cLD and the nLD) are characterized by a median diameter of 0.51 μm but a little more than half are small LD. The size distributions of the two LD classes are: cLD, 57% small > 27% medium > 16% large; nLD 54% small ~ 42% medium > 4% large (Fig 3 and Fig 4). The cytosolic-LD population consists in larger LD than the nuclear, since the respective cLD and nLD maximum-diameter (d_max_) values are 2 and 1.3 μm.

Because of the larger number of cLD than nLD, the total volume of the cLD population per cell (15.8 μm^3^) is greater (by 10.3 times) than that of the nLD (1.53 μm^3^; Fig 4C).

As expected, the results here from primary cultures of rat hepatocytes are comparable to those obtained previously in the laboratory in a rat-liver squash, and in nuclear and subnuclear fractions [2], where a quartile analysis of LD diameters indicated a distribution with a positive skew, owing to a high proportion of LD with a diameter smaller than the median value (*i*. *e*., the small LDs).

#### HepG2 cells

**Fig 5:**
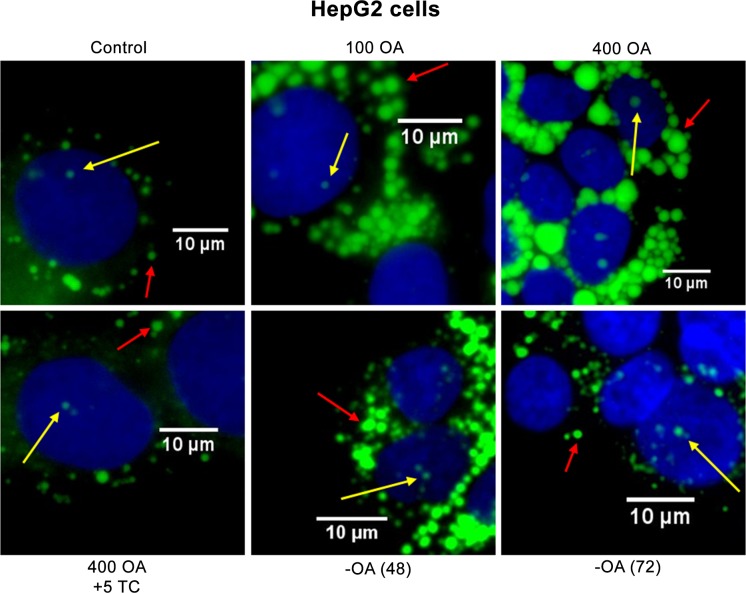
Analysis of cLD and nLD populations of HepG2 cells by fluorescence microscopy. Nuclei (N) and lipid droplets (nLD and cLD) were stained with DAPI (blue) and BODIPY 493/503 (green), respectively. The red and yellow arrows indicate the cLD and nLD, respectively. Control, cells cultured under control conditions for 24 h; 100 OA, 400 OA, cells treated with 400 μM OA for 24 h; 400 OA + 5TC: cells treated with 400 μM OA plus 5 μM TC for 24 h;–OA (48) and–OA (72), cells treated with 400 μM OA for 24 h and then incubated in the absence of OA for 48 or 72 h, respectively. The photographs correspond to representative observations.

**Fig 6:**
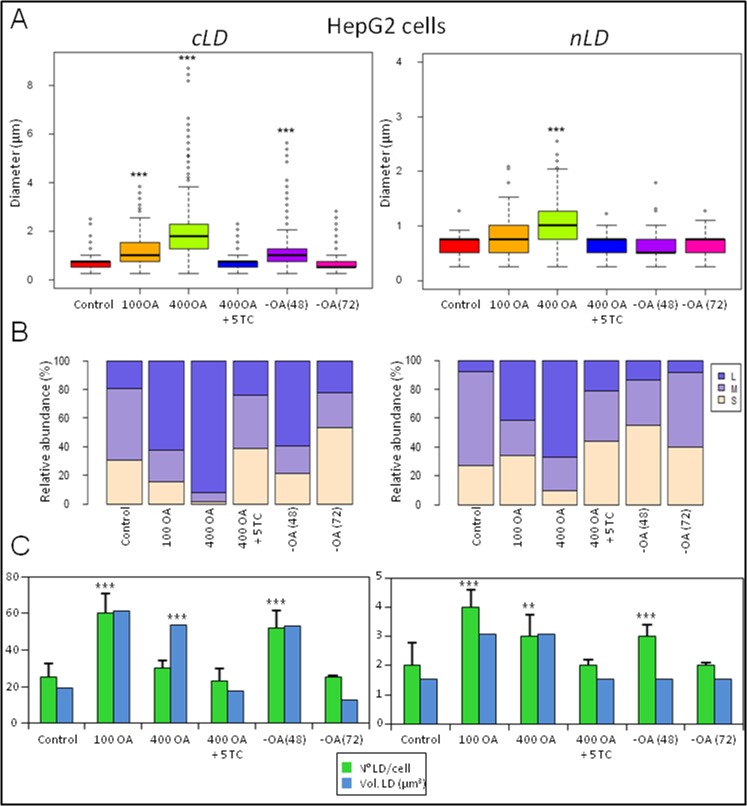
Analysis of the cLD and nLD morphologic parameters of HepG2 cells. HepG2-cell LD populations (cLD and nLD) were measured in fluorescence-microscopy images (equivalent to Fig 5) through the use of Image-Pro Plus software as described in Fig 4. To compare the LD size distribution, the data were analyzed by multiple significance tests with the Bonferroni correction (see S3 and S4 Tables). The experimental groups were the same as for the rat-liver hepatocytes except that this experiment also included a group of cultures treated with 100 μM OA and the incubations in the presence of 1 and 2.5 μM TC were excluded.

The HepG2 cell in culture under control conditions has a mean of 25 cLD in the cytoplasm and 2 nLD in the nucleus, with a median diameter of 0.77 μm (Fig 5 and Fig 6A) for both types of LD and a population composed mainly of medium-sized LD. The size distributions of the two LD classes are: cLD, 50% medium > 31% small > 19% large; nLD, 65% medium > 27% small > 8% large (Fig 6B).

As with the rat hepatocytes, the LD population in the cytosol is larger than in the nucleus since the respective d_max_ values for the cLD and nLD are 2.5 and 1.3 μm.

Because of the larger number of cLD than nLD, the total volume of cLD per cell (19.1 μm^3^) is greater (by 12.5 times) than that of the nLD (1.53 μm^3^) (Fig 6C).

A cellular coordination mechanism must therefore exist in the LD metabolism of these cells such that most of the LD in both populations (the nLD and cLD) have similar sizes (nLD_median_ = cLD_median_). Moreover, the size distribution of the two LD populations (the nLD and cLD) would appear to be characteristic of a given cell type since in hepatocytes most of the LD are small whereas in the HepG2 cells they are medium-sized. The largest LD size (*i*. *e*., indicated by the d_max_) is therefore a distinctive characteristic of each class of LD (nLD or cLD) in both cell types. The nLD population of either cell type, measured as a total volume, represents a small proportion (only 7–9%) of the combined volume of both LD (*i*. *e*., that of the nLD + the cLD) in the cell.

### Effect of OA on the LD morphologic parameters in rat hepatocytes and HepG2 cells

In order to test our hypothesis that the nLD are a dynamic nuclear domain that responds to stimuli external to the cell, we first attempted inducing the enlargement of the nLD by the addition of OA since OA had been implicated in both the genesis and the modulation of cLD [12,13]

#### HepG2 cells

**Fig 7:**
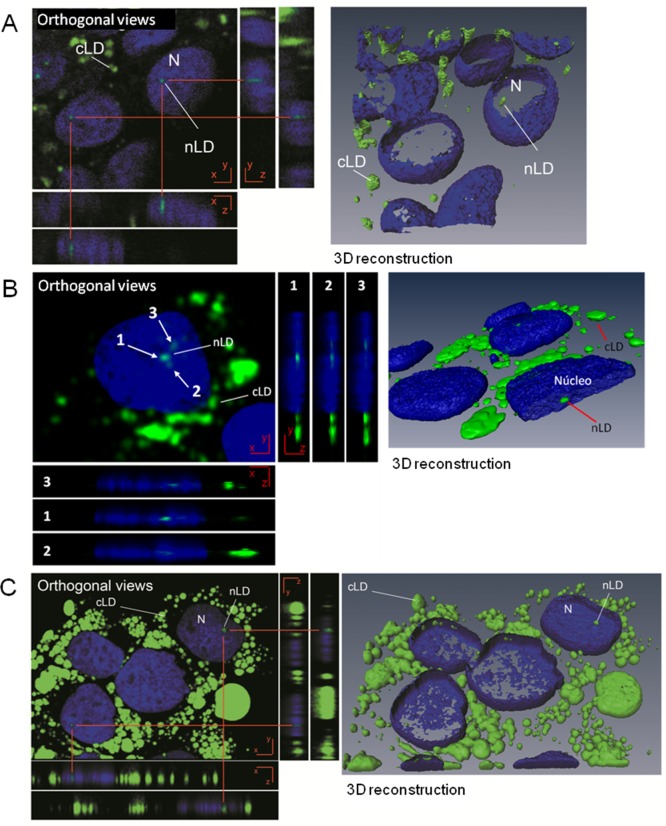
Analysis of HepG2 cells treated with OA by confocal laser-scanning microscopy. (**A**) HepG2 cells cultured under control conditions; (**B**) treated for 24 h with 100 μM OA, (**C**) treated for 24 h with 400 μM OA. For microscopical analysis the nucleus (N) and nLD and cLD were stained with DAPI (blue) and BODIPY 493/503 (green), respectively. Z-plane cross sections through the central position of a nucleus were obtained and the different planes reconstructed to give XZ and YZ orthogonal projections. Three-dimensional reconstructions were performed and the LD visualized through isosurface rendering by BODIPY and outline rendering by DAPI. The photographs correspond to representative observations.

As a first step in optimizing the experimental protocols, the HepG2 cells were treated with OA at different concentrations. When HepG2 cells were cultured with 100 μM OA Figs 5–7, the two LD classes increased both in size—the respective cLD and nLD median diameters being 1.02 and 0.77 μm and the d_max_ values 3.8 and 2.1 μm (Fig 6A)—and in number—at 60 and 4, for the cLD and nLD, respectively. These changes were reflected in an increase in the total LD volume relative to control values (*i*. *e*., 3 and 2 times larger for the cLD and nLD populations, respectively; Fig 6C).

The increase in the number of nLD and cLD after exposure to 100 μM OA may result from the growth of existing smaller LD that reach the threshold of visibility under the microscope and/or from the genesis of LD *de novo*.

When the OA concentration was increased to 400 μM (Fig 6), the increase in the size of both types of LD was dramatic: the respective median diameters of the cLD and nLD became 1.79 and 1.02 μm, the d_max_ values 8.7 and 2.6 μm (Fig 6A), and the total LD volumes 3- and 2-fold larger than the corresponding control volumes (Fig 6C). These results are in agreement with the literature data since in human-hepatoma cells the diameters of the cLD were found increase from 0.54 to 1.9 μm in response to a 24-h treatment with a mixture of palmitic and oleic acids at 500 μM each [14].

In HepG2 cells OA treatment (at 100 and 400 μM) produced an increase in the proportion of large LD in both LD populations (the cLD and nLD) relative to the control values that became greater at the higher OA concentration (Fig 6B): In cells treated with 400 μM OA, 92 and 67% of the cLD and nLD, respectively, fell into the large size category.

In addition, 100 μM OA produced an increase in the number of cLD and nLD with respect to the control values, whereas treatment with 400 μM OA reduced the number of LD in cells to values similar to those of the controls. In contrast, the increase in LD size upon exposure to 100 μM OA was less than that of the cells treated with 400 μM OA. Therefore, the total volume of the cLD and the nLD populations was found to be similar between the two test conditions (100 and 400 μM OA). The simplest explanation for these changes is that 400 μM OA induced an interaction between the small LD that finally generates larger LD (with a greater median diameter and d_max_), thus also producing, as a consequence, a decrease in the LD number. The possible interactions of LD are illustrated in S2 Fig where pairs of nLD and cLD have aggregated in the nucleus and in the cytosol, respectively, after treatment of the cells with 400 μM OA.

HepG2-cell viability was not affected by any of the following supplements to the media: a) BSA (the OA vehicle), b) ethanol (OA dissolution solvent) and c) OA at 100 or 400 μM (S3 Fig).

Since the nLD in the nucleus, like the cLD in the cytoplasm, are a dynamic domain that is increased by OA in both number and size, the next question to answer was whether or not the effect of OA was reversible and the original control LD phenotype could be restored. Accordingly, HepG2 cells and hepatocytes were seeded under control conditions for 48 h, then the medium was replaced by one with 400 μM OA. After a further 24 h, the OA was removed and the cells incubated without OA for 24, 48, or 72 h, as detailed in Fig 1. In both cell types after a 24-h incubation without OA [group–OA (24)], no significant changes were observed in any of the morphologic parameters evaluated, whereas by 48 [group–OA (48)] and 72 h [group–OA (72)] after OA removal significant alterations occurred in the state of the cells with respect to their previous condition in the presence of oleate, as described and discussed below.

In the HepG2 cells, the increases in size and number of the LD induced by 400 μM OA treatment were completely reversed upon exclusion of the OA from the culture medium for 72 h since the morphologic parameters corresponding to those distributions in the two LD populations became comparable to those of the respective control cultures (Figs 5 and 6). In cells incubated in the absence of OA for 48 h, however, the parameters evaluated in the cLD were only partially reversed. In the–OA (48) cultures, the volume of the cLD population was not reduced from the 400 OA value; but the morphologic characteristics were modified, with a greater number of smaller cLD being formed (median diameter, 1.02 μm; d_max_, 5.6 μm; total volume, 53 μm^3^ with a size-class distribution of 60% large > 21% small ~ 19% medium) compared to 400-OA condition (median diameter, 1.79 μm; d_max_, 8.7 μm; total volume, 54 μm^3^ with a size-class distribution of 92% large >> 6% medium ~ 2% small). We can therefore infer that in the–OA-(48) cultures some of the 30 large cLD that characterized the cytosolic population of the 400-OA condition have generated the 52 smaller cLD present 48 h after OA removal by a mechanism that could have involved LD fission. S4 Fig depicts the possible interactions of cLD where two closely apposed cLD were observed, whose contiguousness could suggest that a fissioning had just occurred.

Furthermore, at 72 h after OA was excluded, the nLD parameters that had been modified by 400 μM OA were largely reversed (Figs 5 and 6). Nevertheless, in the HuH7 hepatoma line, the induction of cLD by OA was reported not to have reverted to control conditions as observed here in the HepG2 cells (Fig 5), even after five days of further incubation without OA [15].

Cell viability—evaluated 48 and 72 h by trypan-blue–dye exclusion after treatment with 400 μM OA—was not affected (S5 Fig).

#### Hepatocytes

Since 400 μM OA had produced the most pronounced phenotypic changes in the LD profiles of HepG2 cells, as already described; the hepatocytes were treated under those same conditions (Fig 3).

OA produced an increase in the number (77 per cell) and size (median diameter, 1.28 μm; d_max_, 5.1 μm) of the cLD, as analyzed in Fig 4A. These changes resulted in a 6-fold increase in the total volume of cLD per cell relative to the control values (Fig 4C) and also led to an increase in cLD content.

With 400 μM OA only a single—much larger—nLD (diameter, 1.28 μm) was observed in the nucleus (Fig 4A), with the total volume of the nLD population remaining similar to the control value (Fig 4C). We can therefore infer that 400 μM OA switches on a mechanism that generates a single large droplet (d_max_: 1.8 μm) from the 3 nLD previously present under the control conditions.

The LD-population size distributions (Fig 4B) changed drastically in hepatocytes treated with 400 μM OA because the cLD and nLD became extremely large (cLD: 79% large > 17% medium > 4% small; nLD: 76% large > 22% medium > 2% small). In hepatocytes, the total volume of the nLD population thus remained statistically unchanged, whereas the number and size distribution changed markedly upon OA treatment.

The decreases in the nLD population produced by 400 μM OA were reversed when the OA was excluded from the culture medium for 48 or 72 h since after that transition the morphologic parameters and size distribution of the nLD became similar to those of the control values (Fig 4).

In Group–OA (48), the volume of the cLD population was initially not greatly modified, but the morphologic characteristics were rearranged since a greater number of smaller cLD were formed (median diameter, 0.51μm; d_max_, 4.3 μm; total volume, 62.8 μm^3^; 51% small > 26% medium ~ 23% large) compared to those respective parameters under 400-OA conditions (median diameter, 1.28 μm; d_max_, 5.1 μm; total volume, 98.2 μm^3^, 79% large > 17% medium > 4% small; Fig 4B). Since the cLD total volume in the–OA (48) group was not statistically different from the 400 OA value, we can infer that in Group–AO (48), some of the 77 cLD present in the cytosol after treatment with 400 μM OA, underwent a mechanism that generate the 123 smaller cLD (Fig 4C). Unlike what was observed with the HepG2 cells, in the hepatocytes 48 h after OA removal the cLD size distribution and median diameter of the control conditions became reestablished (Fig 4A).

At 72 h after treatment with 400 μM OA, the hepatocytes cLDs' size characteristics (median diameter, 0.51 μm; d_max_: 2.5 μm) and size distribution (54% small > 31% medium > 15% large) became restored to the control values (Fig 4A and 4B) but with 3 times the number of cLD being present in the cytosol—*i*. *e*., 107 as opposed to 31—so that the total volume of cLD was greater than under the control conditions (Fig 4C), being barely decreased below the level in the presence of 400 μM OA (Fig 4C). Unlike this response in the hepatocytes, in the HepG2 cells, the number of cLD became restored to control values at 72 h after OA was removed.

#### Summary of the characteristics of the LD morphologic changes effected by OA

The following experimental evidence would indicate that nLD are a dynamic nuclear domain that responds to external stimuli when compared to physiological control conditions.

1 nLD and cLD populations increase in size in response to extracellular OA through a mechanism that determines that both populations of LD (cLD and nLD) became composed mainly of large LD.
2 Cellular LD metabolism is coordinated according to the subcellular compartment: the LD of HepG2 cells and hepatocytes are organized such that the nLD and cLD populations are composed of mainly medium and small LD, respectively, as a phenotypic characteristic of the two populations under control physiological conditions.

In this regard, since the morphologic changes induced by OA in the nLD and cLD populations that produced the larger LD (*i*. *e*., the d_max_) were greater in HepG2 cells than in hepatocytes, we can consider that hepatocytes constitute a more highly regulated hepatic model than the hepatoma cell line.

3 The morphologic changes with respect to LD population size effected by OA are more stringent in the nucleus than in the cytosol. The increases in size induced in the nLD and cLD by OA became reverted when OA was excluded from the medium—with, however, response characteristics that were peculiar to each cell type. In HepG2 cells the LD morphologic parameters that had been augmented by OA were fully reversed when the FA was excluded from the medium since the nLD and cLD returned to a phenotype similar to that of the control cells 72 h later.
4 The effect of OA on LD morphology is reversible, but occurs with characteristics peculiar to each class of LD as well as to each type of cell. Although in hepatocytes—in contrast to HepG2 cells—the median diameter and the size distribution of the cLD and nLD had already recovered control values by 48 h after OA removal, the number of cLD remained increased, though the beginning of a decline seemed to be observed, whereas nLD number had dropped to control values.

### Effect of OA on cellular-lipid composition

**Fig 8:**
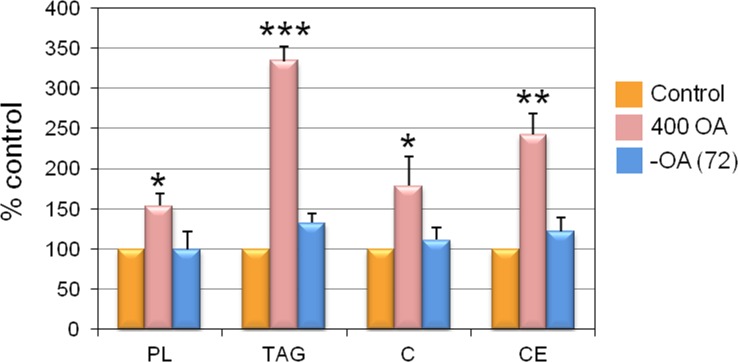
Lipid composition of HepG2 cells. The results for each experimental condition—calculated as a percent of the respective control values and plotted on the *ordinate* for each lipid class indicated on the *abscissa*—are presented as the means ± SD of 3 independent experiments expressed as nmol *per* 10^6^ cells of each lipid class as calculated on the basis of the following molecular weights: PL, 796; TG, 864; C, 387; and CE, 641. *p <0.05, **p <0.01, ***p <0.001. Experimental groups: 400 OA, HepG2 cells cultured in the presence of 400 μM OA for 24 h;–OA (72), 400 OA cells cultured for 72 h after OA removal. Since the lipid spectrum of this permanent cell line may change during each culture passage, we needed a nonstimulated control for a given experimental condition at each time point. Therefore, the control for Group 400 OA was unstimulated cells after 72 h in culture and for the Group–OA (72) parallel cultures without treatment for 144 h (*cf*. Fig 1. Accordingly, the lipid composition of the control for Group 400 OA was PL = 6.8 ± 0.1, TAG = 5.0 ± 1.4, C = 3.8 ± 1.0, CE = 0.8 ± 0.3and for Group–OA (72) was PL = 11.6 ± 2.1, TAG = 3.4 ± 1.9, C = 12.7 ± 2.9, CE = 2.6 ± 0.4.

In order to determine if the reversible effects on the nLD and cLD by OA also involved changes in the cellular-lipid classes, HepG2-cell lipids—composed of PL (42%) along with the neutral lipids (NL) TAG, C, and CE (58% total)—were quantified under the different experimental conditions iterated above (Fig 8).

In HepG2 cells treated with 400 μM OA all the lipid classes tested increased above the control values by the following increments (Fig 8): PL (54.2%) and the NL: TAG (234%), C (78.6%) and EC (143%). In contrast, after 72 h after the removal of OA from the medium—*i*. *e*., Group–OA (72)—the increments in the PL as well as in the NL were reversed, making the content of each lipid class become comparable to the corresponding control value. Throughout these experiments, the cellular protein content was unchanged under all the conditions evaluated (S6 Fig).

In HuH7 cells, however, TAG and CE were reported to have increased in response to OA, whereas C remained unchanged in this hepatoma cell line [15].

We can consider that cellular NL are mainly localized in the cLD since the nLD only corresponded to 2–10% of the cellular-LD population (by volume) and those nLD represented only 0.002% of the cellular lipids [2]. Moreover, to determine the chemical composition of the nLD, an initial isolation of the nuclei of the cultured cells would be necessary followed by an isolation of the nLD before the respective biochemical measurements could be made. Because the final yields after the whole isolation process would be prohibitively low, to make such determinations would not be currently possible. Nor could the corresponding compositions of the cLD and nLD isolated from hepatocytes in primary culture be realized since those cells are grown in even smaller quantities. In order to make such determinations, much greater numbers of cells would be required for both types of cultures. We believe, however, that the biochemical composition of primary hepatocyte cultures under control conditions would nevertheless be equivalent to those previously obtained in the laboratory from the nLD and cLD isolated from rat-liver nuclei and homogenates, respectively [2].

In conclusion, the addition of OA to HepG2 cultures produced an increase in the PL and NL content that was reversed when OA was removed from the medium (–OA). The classes TAG and CE in particular—those being located mainly in the cellular LD [2]—were the lipids most greatly increased by exposure to OA; and this elevation was, in turn, reflected in the greater number, size, and volume of the LD present. Because the PL and C are components of all cell membranes as well as being constituents of the LD monolayer, the increments induced by OA in those lipid classes would reflect increases in cell membranes in general.

### Involvement of lipid synthesis in the OA-induced increases in lipid classes

In order to determine whether a synthesis of PL and NL was involved in the OA-induced increases in the nLD- and cLD-associated lipids, cells were cultured with TC, an acyl-CoA-synthetase inhibitor [16]. TC inhibits the synthesis and remodeling of PL and NL, by blocking the formation of acyl-CoA, a substrate indispensable to this process [16].

#### HepG2 cells

**Fig 9:**
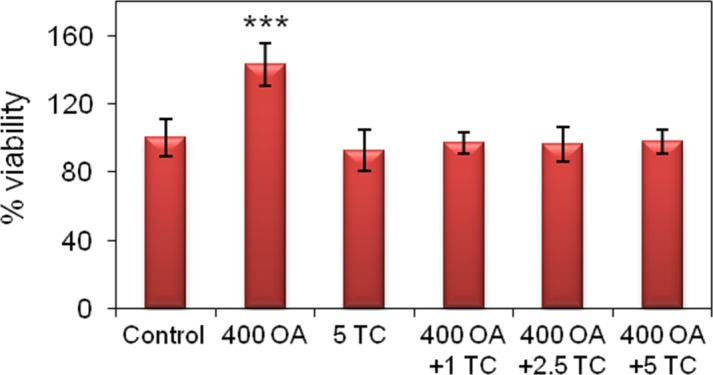
HepG2-cell viability after treatment with OA and the lipid-synthesis inhibitor Triacsin C. Cell viability was determined by the MTT assay. The data are expressed as the mean ± SD of 3 independent experiments (n = 3); *p <0.05, **p <0.01, ***p <0.001. Experimental groups: Control, cells incubated under control culture conditions; 400 OA, cells treated with 400 μM OA for 24 h; 400 OA + 1 TC, cells treated with 400 μM OA plus 1 μM TC for 24 h; 400 OA + 2.5 TC, cells treated with 400 μM OA plus 2.5 μM TC for 24 h; 400 OA + 5 TC, cells treated with 400 μM OA plus 5 μM TC for 24 h. In the figure, the percent viability is plotted on the *ordinate* for the experimental groups indicated on the *abscissa*.

HepG2 cells were incubated with 400 μM OA plus TC at 5 μM, the concentration generally used in the literature for this purpose (Fig 1) [17]. When cell viability was measured by the MTT assay in the presence of increasing concentrations of TC (1, 2.5, and 5 μM; Fig 9), no decrease was observed relative to the control values at any of the concentrations of TC used. In contrast, exposure to 400 μM OA resulted in a significant increase relative to the control condition. This elevation can be attributed to the enhancement in cellular metabolism produced by OA that has been reported [18]. Notwithstanding, the cellular-protein content (in mg *per* 10^6^ cells) remained constant under these experimental conditions (S6 Fig), as did cell viability as determined by cell counting (S5 Fig).

The increase in lipids induced in HepG2-cell cLD and nLD by OA was inhibited by 5 μM TC treatment since the LD morphologic parameters (*i*. *e*., median diameter, d_max_, and cell number and volume) of both populations of LD remained comparable to the control values (Fig 6).

#### Hepatocytes

Considering that rat primary hepatocytes are highly sensitive to culture conditions, we first evaluated the optimum concentration of TC that totally inhibited the effect of 400 μM OA and at the same time did not decrease cell viability. For this purpose, hepatocytes were incubated with 400 μM OA with the following increasing concentrations of TC: 1, 2.5, and 5 μM (Fig 4).

**Fig 10:**
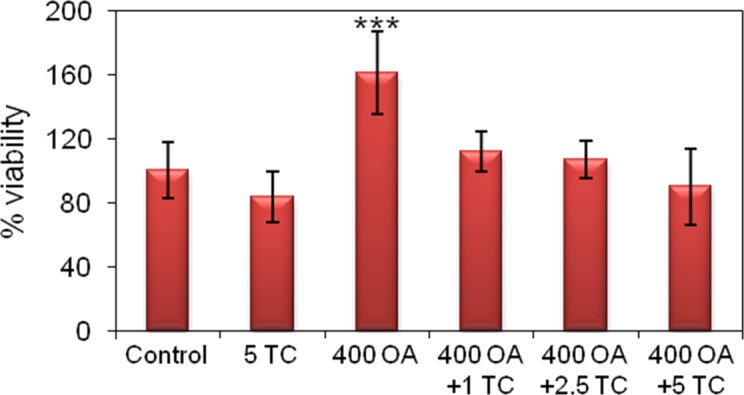
Cell viability of rat primary hepatocytes treated with OA and Triacsin C inhibitor. Cell viability was determined by the MTT assay. The data are expressed as the mean ± SD of 3 independent experiments (n = 3); *p <0.05, **p <0.01, ***p <0.001. Experimental groups: Control: cells incubated under control culture conditions; 400 OA, cells treated with 400 μM OA for 24 h; 400 OA + 1 TC, cells treated with 400 μM OA plus 1 μM TC for 24 h; 400 OA + 2.5 TC, cells treated with 400 μM OA plus 2.5 μM TC for 24 h; 400 OA + 5 TC, cells treated with 400 μM OA plus 5 μM TC for 24 h. In the figure, the percent viability is plotted on the *ordinate* for the experimental groups indicated on the *abscissa*.

Hepatocytes-cell viability as determined by the MTT assay underwent no significant changes as a result of TC treatment compared to the control values (Fig 10). As observed in HepG2 cells, a significant increase in the measurement occurred in the hepatocytes treated with 400 μM OA relative to the control data, likewise attributable to a general increase in cellular metabolism [18]. These results are in agreement with literature data [19].

Next, the treatment of hepatocytes with 400 μM OA plus TC revealed that the high percentage of large cLD and nLD characteristic of both LD populations treated with 400 μM OA became progressively diminished as a function of the concentration of TC added to the medium (Fig 4B); with values in cells treated with 400 μM OA plus 5 μM TC reaching cLD and nLD size distributions similar to those of the respective control populations, where both LD classes are characterized by a composition consisting of mostly small droplets. The other morphologic parameters analyzed in the cLD and nLD also became normalized as a function of the TC concentration, reaching control values in cells treated with 400 μM OA plus 5 μM TC (Fig 4A and 4C).

We conclude that OA induces alterations in nLD and cLD through a dynamic, reversible, and coordinated process requiring ongoing lipid biosynthesis.

## Discussion

In this report we have demonstrated that nLD are a dynamic nuclear domain that is modified by the presence of OA in terms of the number and size of the nLD population, through a reversible mechanism that involves acyl-CoA–synthetase activity in the biosynthesis of TAG, CE, and PL. These results would imply that nLD would be actively involved at least in those processes taking place within the nucleus that include lipids.

The LD constitute the subcellular organelle where NL are stored and compartmentalized at a supramolecular level with the location of those lipids being principally in the cLD of the cytosol and involving only a small proportion in the nLD of the nucleus [2]. A form of cellular coordination in NL metabolism must necessarily exist between the those two LD population that was reflected in the same qualitative morphologic changes observed in both the nuclear- and the cytosolic-LD populations under all conditions tested, thus insuring that the nLD always corresponded to a small proportion (2–10% by volume) of the total cellular LD (*i*. *e*., the nLD plus the cLD).

An interdependence has been reported to exist between the NL metabolism from a host cell and both bacterial (*Mycobaterium tuberculosis*) [20,21] and viral (hepatitis-C virus and Dengue) pathogens [22,23]; with the role of cLD having been cited in these processes. An initial consideration of the functioning of not only the cLD but also nLD of the host in infections as well as in hepatic diseases (*e*. *g*., nonalcoholic fatty liver) and in liver regeneration, transplantation, and organoids [24] would certainly be relevant. Under physiologic, nonpathologic, and unstimulated conditions the nLD could be analyzed and quantified in rat-liver squashes and in both primary cultures of hepatocytes and HepG2 cells [2].

The selection of the appropriate experimental cellular model is always essential. When studying liver-lipid metabolisms, the cell line used should have nLD even under physiologic, nonpathologic, and unstimulated conditions, as observed in both rat hepatocytes and HepG2 cells (Fig 2) [2]. nLD could be considered as a liver marker for the control—*i*. *e*., physiologically normal—cell phenotype. nLD would not, however, be present in all cell models since such droplets were not observed in even OA-treated HeLa, HF34, Y1, and OP9 cells [3]. In contrast, in the Huh 7 line, nLD were present in OA-treated cells as well as after promyelocytic leukemia nuclear bodies -isoform-II manipulations [3]. In that liver-cell model, the morphologic induction of cLD by OA would involve an irreversible mechanism since the cLD control phenotype could not be reestablished even after five days of further cell incubation without OA [15], with those results being unlike what was observed here in the rat hepatocytes and HepG2 cells (Figs 3 and 4). Finally, in yeast, nLD were detected in the mutant (*Ldb16*) but not the wild-type cells, and not even after OA supplementation [25].

Nevertheless, the nLD morphologic changes in the nuclei of liver cells, human cell lines (*e*. *g*., HepG2), and rat hepatocytes were of a lower magnitude than those of the cLD, possibly owing to a more highly regulated nuclear metabolism of NL and/or a reduced availability of nLD proteins and/or lipids compared to that of the cLD.

In addition, we can consider that the hepatocyte LD metabolism involves a different kinetics of LD morphology than that of HepG2 cells since even though both types of LD were dramatically affected by OA, the morphologic changes in the hepatocytes were reversed in a shorter time, perhaps because the largest nLD and cLD (d_max_) in the hepatocytes were smaller than those of the HepG2 cells. Moreover, previous findings had already determined that although lipid synthesis was very active in HepG2 cells, those cells harbored a defect in lipolysis and lipid oxidation and also secreted lipoproteins [26], which characteristics would explain the greater time needed to restore the LD control phenotype (smaller nLD and cLD populations) than required by normal hepatocytes when OA had been excluded. An alternative consideration would be that because in adipose tissue the smaller LD are metabolically more active than the larger ones [27], the smaller LD populations in the hapatocytes would be more metabolically active than those in the HepG2 cells.

In this work we have demonstrated that liver cells, in response to external stimulation by OA, exhibit a net activation of anabolic pathways that produces an increase in the number and size of nLD and cLD through the incorporation of lipids that have been synthesized *de novo* (*i*. *e*., TAG, CE, and PL).

OA has been found to induce cellular metabolism and also increases the expression of the peroxisome-proliferator–activated receptor α (PPAR α) in liver [28]. By this mechanism, liver steatosis induced by OA would be prevented by promoting FA oxidation in mitochondria and peroxisomes [29]; an increase in the reactive-oxygen species induced by OA has also been reported [18].

The anabolism triggered by OA that produced the dramatic induction of nLD and cLD in both the liver-cell models tested was reversed by a net balance of catabolic pathways that were brought into play by the elimination of the FA from the culture medium and that also involved a mechanism that transformed larger nLD and cLD into smaller ones.

These catabolic processes would include cellular lipolysis and the subsequent mobilization of hydrolyzed FA from the LD to cytosolic-oxidation sites in the mitochondria and peroxisomes and/or the exportation of those FA as VLDL particles [26,30]. These processes would explain the cellular lipid (TAG, CE, C and PL) reduction as well as the smaller size of the LD observed in both populations after OA removal. Within this context, the cellular NL are organized in the form of cLD and nLD as has already been reported [2]. For example, the nuclear DAG and MAG lipases could be involved in nLD lipolysis [31], while the FA-binding protein (FABP) would mobilize FAs within the nuclear matrix [32]. The PL would also be hydrolyzed from the nLD monolayer by the nuclear phospholipases A2 (PLA2), C, and D [33]. Moreover, the phosphatidic acid (PA) generated in the nucleus by the action of the OA-induced phospholipase D (PLD) [33] could generate crucial second messengers involved in these processes [31].

Nuclear bodies—and particularly speckles, paraspeckles and Cajal bodies—have also been shown to be dynamic nuclear domains that respond by altering their number and size during the cell cycle and in response to different stimuli [1]. The local recruitment of molecules—mainly proteins and nucleic acids—has been proposed to be one of the mechanisms involved in the morphologic dynamics of nuclear bodies [34]. Since nLD are the only nuclear domain composed of lipids and surrounded by a lipoprotein monolayer, the increase in the nLD in both size and number must be a complex and highly regulated cellular process that is both coordinated with the cLD and different from that of the nuclear bodies. Under control physiologic conditions, nLD are not found in any particular spatial relationship with the nuclear bodies speckles, paraspeckles, and nucleoli or with lamin A or the lamin-binding peptide 2ß (LAP2ß) [2]. OA has been reported to induce nuclear-membrane invaginations (type-I and/or type-II nucleoplasmic reticulum) [35]. Accordingly, in OA- and DHA- stimulated Huh7 cells, nLD have been found to be associated with promyelocytic leukemia nuclear bodies and type-I nucleoplasmic reticulum [3]. Moreover, in the livers of mice fed a high-fat diet, nLD were also reported to be associated with promyelocytic leukemia nuclear bodies nuclear bodies [3].

In eukaryotic cells different models have been proposed to explain the mechanism of cLD generation from the endoplasmic reticulum (ER) within the cytosol, among which schemes the budding model is the one most widely cited [36,37]. Indeed, nLD could be generated in the cytosol and/or the nucleus as follows: 1) after generation in the ER, small immature LD could enter the nucleus through the nuclear-pore complex and/or be trapped in the nucleus after mitosis when the nuclear membrane was formed. The LD originating in the ER would have to mature since nLD have a chemical composition different from that of the cLD in terms of individual lipid-class proportions and moreover do not contain the perilipin-1 (PLIN 1) and acetyl-CoA–acetyltransferase-1 (ACAT 1) proteins under physiological control conditions [2]. 2) A generation in the inner nuclear membrane could occur through a mechanism similar to that of cLD formation in the ER since the nuclear membrane has spatial continuity with the ER. Particularly in Huh7 cells stimulated with OA, promyelocytic leukemia nuclear bodies and nucleoplasmic reticulum have been proposed to be involved in nLD formation [3]. In prokaryotic organisms such as the *Rhodococcus* bacteria, cytoplasmic LD are generated in the plasma membrane [38]. Moreover, a correlation between plasma-membrane caveolae and cLD dynamics has been reported in adipose tissue [39].

It is not well understood the mechanism by which nLD morphology changes by increasing the LD number and size of the population, but the following mechanisms have been proposed for LD enlargement: 1) *LD Fusion*: That LD fusion is a widespread cellular mechanism is widely accepted, as the occurrence has been described in the cytosol of cells from eukaryotes as well as bacteria [38,40] According to data from the literature, in all fusion processes between two vesicles (with bilayers), two LDs (with monolayers), and an LD with a bilayered vesicle; SNARE (soluble N-ethylmaleimide-sensitive–factor activating receptor) proteins among others are involved [14]. For example, cLD fusion in stimulated *Rhodococcus* bacteria takes place through a mechanism involving "SNARE-like proteins" [37,38]. To the best of our knowledge, SNARE proteins have not been described in the cell nucleus. nLD from Huh7 cells stimulated with OA and/or docosahexanoic acids, however, were observed to be interacting through physical associations, which contiguities were interpreted by transmission electron microscopy as fusions [3]. 2) *cLD* contact sites: CIDE proteins located in contact sites between to cLD that are interacting would promote and control lipid exchange as well as transfer along with LD fusion and growth in both hepatocytes [41] and adiposites [42]. A transfer of net lipids from the smaller to the larger LD via contact sites in the LD monolayer has been proposed 3) *Lipids locally synthesized in the cLD*: The enlargement of preexisting cLD would be generated by the synthesis of PC and TAG mediated by CCTα and GPAT, respectively, present in the LD monolayer [43].

That during the differentiation of adipocyte cells cLD morphologic changes could be explained by a combination of different processes such as LD fusion and the incorporation of lipids into preexisting cLD has been proposed; and if catabolism is stimulated, cLD fission would reduce the LD size along with lipolysis and the interaction of LD with other cytosolic organelles [44]. That in adipocye cells [45] and in *Drosophyla* [46] a fragmentation and dispersion of large into smaller cLD involving perilipins has also been reported.

We can consider that the increase in LD size and number by OA treatment of liver cells (Figs 2 and 3) could result from the incorporation of TAG synthesized in the nucleus since in OA-stimulated Huh7 cells diacylglycerol–O-acyltransferase 2 (DGAT2) colocalizes with the nLD and cLDs [3]. Moreover, nuclear acyl-CoA-synthetase, which enzyme catalyzes fatty-acid activations to produce acyl-CoAs [47], would provide the substrates for nuclear TAG biosyntheses. CCTα involved in phosphatidyl-choline (PC) synthesis, translocates between the inner nuclear membrane and speckles in response to specific stimuli [48]. Moreover, OA can induce CCTα translocation to the surface of cLD and nLD, thus providing the necessary PC in the monolayer coating to generate larger LD [3,49]. These mechanisms would explain the increases in PL observed in liver cells after OA treatment (Fig 8).

nLD proteomic data along with marker proteins are still lacking. An identification of nLD proteins would allow us to understand the cellular processes in which nLD are involved as well as the genesis of nLD. Nevertheless, under control conditions the cLD proteins PLIN 1 and ACAT 1 could not be identified in rat-liver nucleus [3], and only few proteins associated with nLD under stimulated conditions have been localized [3], as discussed above.

Cellular nuclear signaling must be turned on and off, with lipids (FA, DAG, PA, PL) possibly participating in some of the nuclear processes as switching moieties between metabolic pathways. The nLD could be involved in lipid homeostasis and serve as a buffer system capable of providing or incorporating local lipids along with the proteins of lipid metabolism—and of other processes taking place within the nucleus—through a reversible mechanism. nLD-generated lipids could, in turn, be involved in signaling pathways by providing ligands for transcription factors (PPAR, HNF4α, etc.) and/or the substrates of enzymes (CCTα, ACS, delta 5 desaturase, DAG lipase, etc.).

nLDs in particular could provide local FA released by lipolysis and mobilized as ligands of the FABP; FA could bind to transcription factors such as PPARs. Finally, when the process had to be switched off; the FA could be released from the transcription factor, bound to the nuclear FABP, and finally mobilized, esterified, and/or incorporated into nLD lipids. An equivalent process could also involve the other nLD-lipid classes such as DAG, PA, C, phosphatidyl inositols, and sphingolipids.

## Conclusions

nLD are a dynamic nuclear domain reversibly induced by OA that requires the ongoing biosynthesis of TAG, CE, and PL and involves morphologic and size changes within the LD populations. The metabolism of nLDs and cLD is related in a coordinate fashion, with the cellular proportion of the nLD under all conditions tested remaining small, at less than 10% of the total LD (nLD plus cLD). Thus the liver nLD and cLD are both dynamic cellular organelles. The nLD could be considered a liver marker for the control—i. e., normal cell-physiologic—phenotype in the choice of an appropriate experimental model for investigating liver biochemistry.

## Supporting Information

S1 Fig Analysis of primary rat hepatocytes and HepG2 cells by bright-field microscopy. **(A)** Rat primary hepatocytes; below; **(B)** HepG2 cells, both cultured under control conditions. The photographs correspond to representative observations. N, nucleus.
(TIF)

S2 Fig nLD and cLD in close proximity in HepG2 cells. Visualization by fluorescence microscopy of nLD (Panel A) and cLD (Panel B) in close proximity in HepG2 cells treated with 400 μM OA. Nuclei (N) and LD (nLD and cLD) were stained with DAPI (blue) and BODIPY 493/503 (green), respectively. The magnified fields at the right show detailed regions in which the LD in close proximity are outlined by dotted circles.
(TIF)

S3 Fig Cell viability in HepG2 cells treated with OA. Cell viability was determined by cell counting. The data are expressed as the mean ± SD of three independent experiments. The statistical significance of differences among the data was evaluated by ANOVA with *post-hoc* comparisons of the means via Tukey's honestly-significant-difference test (p <0.05). Key to experimental groups: MEM, cells incubated under standard culture conditions; Et+BSA, cells incubated under standard conditions plus ethanol used for OA solubilization and BSA, the OA vehicle; 100 OA, cells treated with 100 μM OA for 24 h; 400 OA, cells treated with 400 μM OA for 24 h.
(TIF)

S4 Fig cLD clost together in HepG2 cells. cLD were visualized in HepG2 cells stimulated with 400 μM OA 48 h (Panel A) and 72 h (Panel B) after OA removal. Nuclei (N) and cLD were stained with DAPI (blue) and BODIPY 493/503 (green), respectively. The magnified fields at the right show detailed regions in which cLD are clost together are outlined by dotted circles.
(TIF)

S5 Fig Cell viability of HepG2 cells after treatment with OA. Cell viability was determined by cell counting. The data are expressed as the means ± SD of three independent experiments. The statistical significance of differences among the data was evaluated by ANOVA with *post-hoc* comparisons of the means via Tukey's honestly-significant-difference test (p <0.05). Key to experimental groups: Control, cells incubated under control culture conditions; 400 OA. cells treated with 400 μM OA for 24 h;–OA (48) and–OA (72), cells treated with 400 μM OA for 24 h and then incubated in the absence of OA for 48 h and 72 h, respectively. In the figure, the percent viability is plotted on the *ordinate* for the experimental groups indicated on the *abscissa*.
(TIF)

S6 Fig Protein content of HepG2 cells treated with OA. The protein content, expressed as mg of protein *per* 106 cells, and cell number were determined under the different culture conditions. The data are the means ± SD for three independent experiments in mg protein *per* 106 cells. The statistical significance of differences among the data was evaluated by ANOVA with *post-hoc* comparisons of the means via Tukey's honestly-significant-difference test (p <0.05). Key to experimental groups: Control, cells cultured under control conditions; 400 OA, cells treated with 400 μM OA for 24 h; and–OA (72), cells treated with 400 μM OA for 24 h and then incubated in absence of OA for 72 h. In the figure, the cellular-protein content in mg is plotted on the *ordinate* for the experimental groups indicated on the *abscissa*.
(TIF)

S1 Table Statistical analysis of the relative abundance—cLD of hepatocytes. The data corresponds to statistical analysis of the cLD size distribution in hepatocytes (Fig 4). Each experimental treatment defined in Fig 4 was compared with the corresponding control condition for the same LD-size category (small, medium, or large; *p<0.05, **p<0.01, ***p<0.001).
(DOC)

S2 Table Statistical analysis of the relative abundance—nLD of hepatocytes. The data corresponds to statistical analysis of the nLD size distribution in hepatocytes (Fig 4). Each experimental treatment defined in Fig 4 was compared with the corresponding control condition for the same LD-size category (small, medium, or large; *p<0.05, **p<0.01, ***p<0.001).
(DOC)

S3 Table Statistical analysis of the relative abundance—cLD of HepG2 cells. The data corresponds to statistical analysis of the cLD size distribution in HepG2 cells (Fig 6). Each experimental treatment defined in Fig 6 was compared with the corresponding control condition for the same LD-size category (small, medium, or large; *p<0.05, **p<0.01, ***p<0.001).
(DOC)

S4 Table Statistical analysis of the relative abundance—nLD of HepG2 cells. The data corresponds to statistical analysis of the nLD size distribution in HepG2 cells (Fig 6). Each experimental treatment defined in Fig 6 was compared with the corresponding control condition for the same LD-size category (small, medium, or large; *p<0.05, **p<0.01, ***p<0.001).
(DOC)
