# Supplementary figures and images for: Reversible Nuclear-Lipid-Droplet Morphology Induced by Oleic Acid: A Link to Cellular-Lipid Metabolism
Source: PLoS One. 2017 Jan 26;12(1):e0170608. doi: 10.1371/journal.pone.0170608 (PMC5268491; doi:10.1371/journal.pone.0170608)

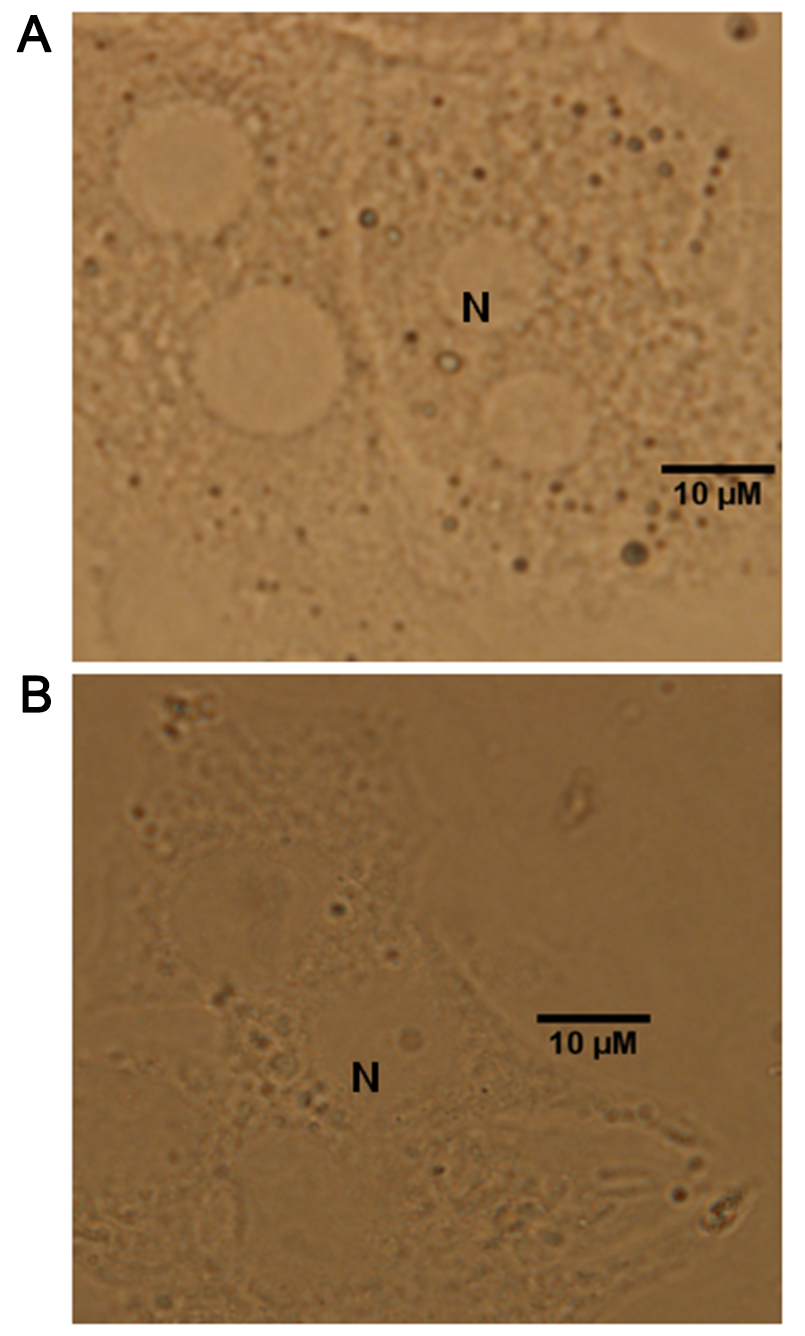

Supplement: S1 Fig — (A) Rat primary hepatocytes; below; (B) HepG2 cells, both cultured under control conditions. The photographs correspond to representative observations. N, nucleus. (TIF) [file pone.0170608.s001.tif]

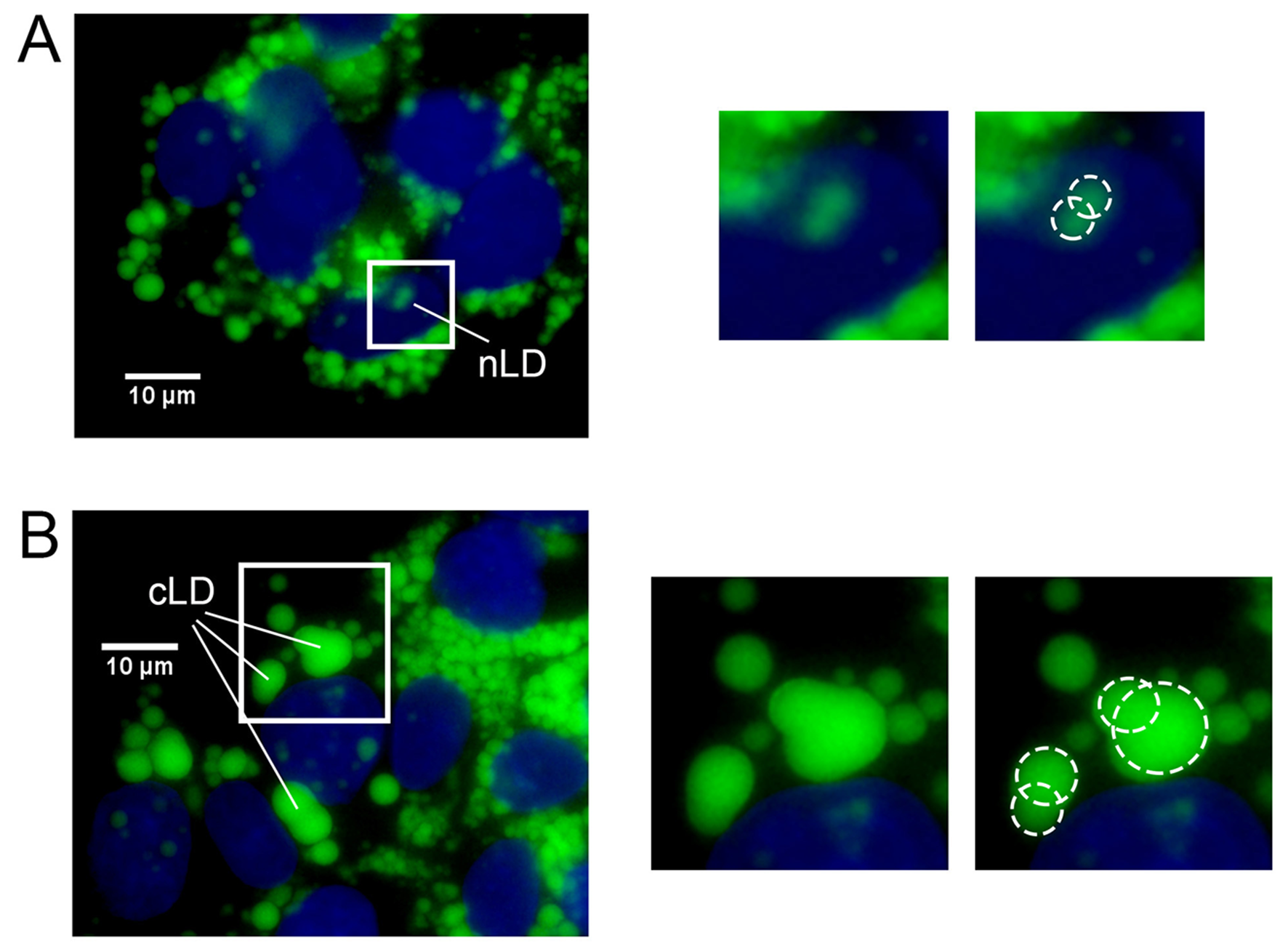

Supplement: S2 Fig — Visualization by fluorescence microscopy of nLD (Panel A) and cLD (Panel B) in close proximity in HepG2 cells treated with 400 μM OA. Nuclei (N) and LD (nLD and cLD) were stained with DAPI (blue) and BODIPY 493/503 (green), respectively. The magnified fields at the right show detailed regions in which the LD in close proximity are outlined by dotted circles. (TIF) [file pone.0170608.s002.tif]

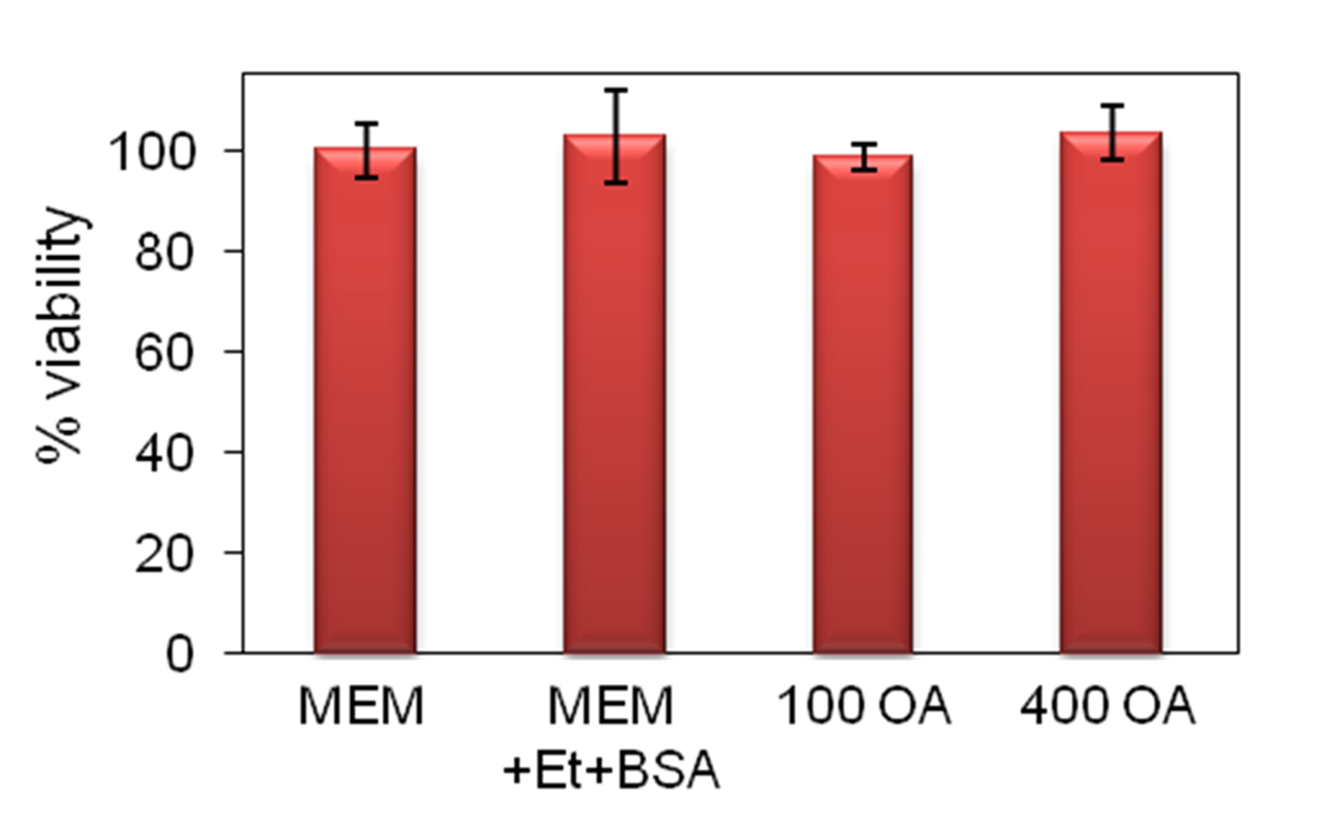

Supplement: S3 Fig — Cell viability was determined by cell counting. The data are expressed as the mean ± SD of three independent experiments. The statistical significance of differences among the data was evaluated by ANOVA with post-hoc comparisons of the means via Tukey's honestly-significant-difference test (p <0.05). Key to experimental groups: MEM, cells incubated under standard culture conditions; Et+BSA, cells incubated under standard conditions plus ethanol used for OA solubilization and BSA, the OA vehicle; 100 OA, cells treated with 100 μM OA for 24 h; 400 OA, cells treated with 400 μM OA for 24 h. (TIF) [file pone.0170608.s003.tif]

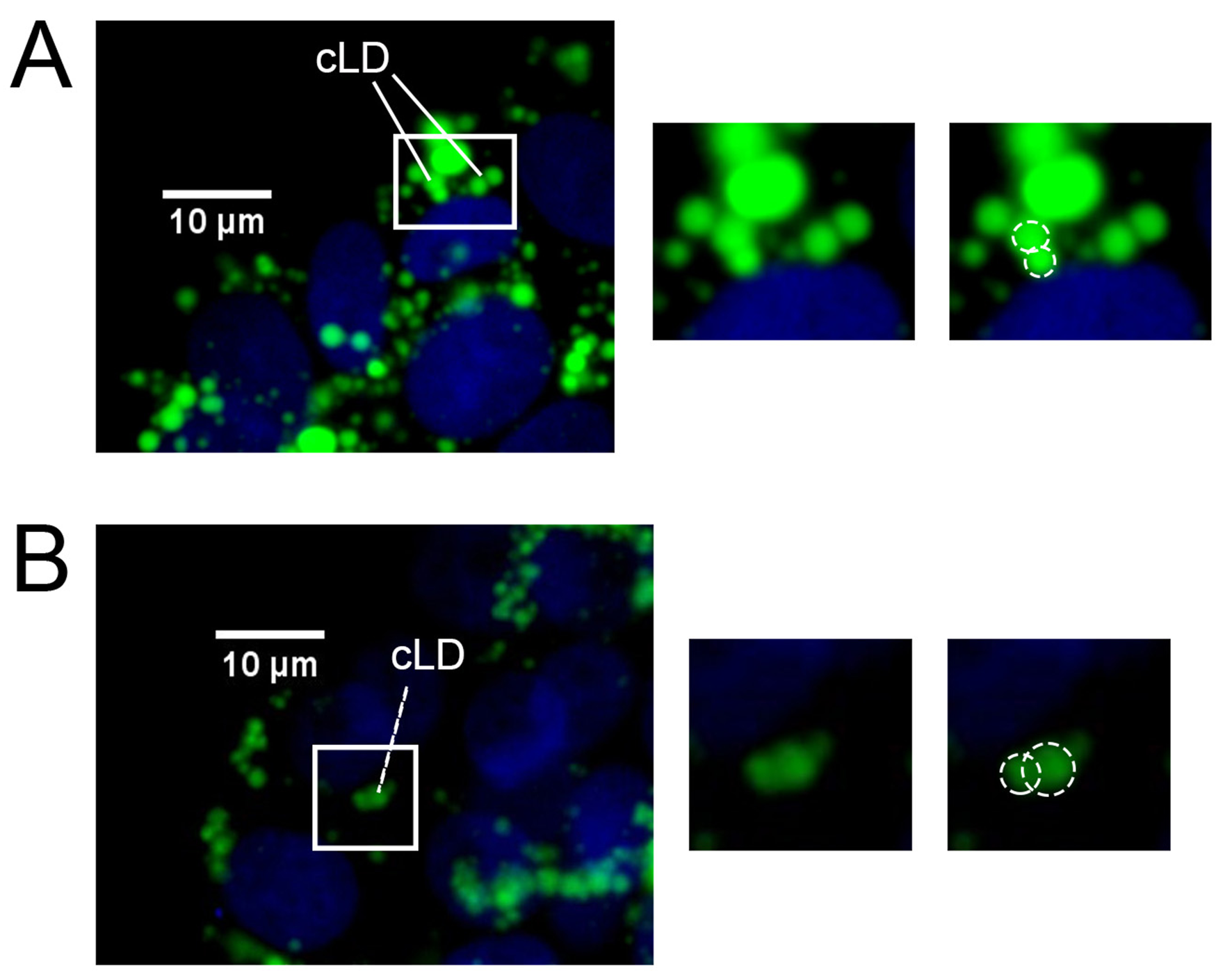

Supplement: S4 Fig — cLD were visualized in HepG2 cells stimulated with 400 μM OA 48 h (Panel A) and 72 h (Panel B) after OA removal. Nuclei (N) and cLD were stained with DAPI (blue) and BODIPY 493/503 (green), respectively. The magnified fields at the right show detailed regions in which cLD are clost together are outlined by dotted circles. (TIF) [file pone.0170608.s004.tif]

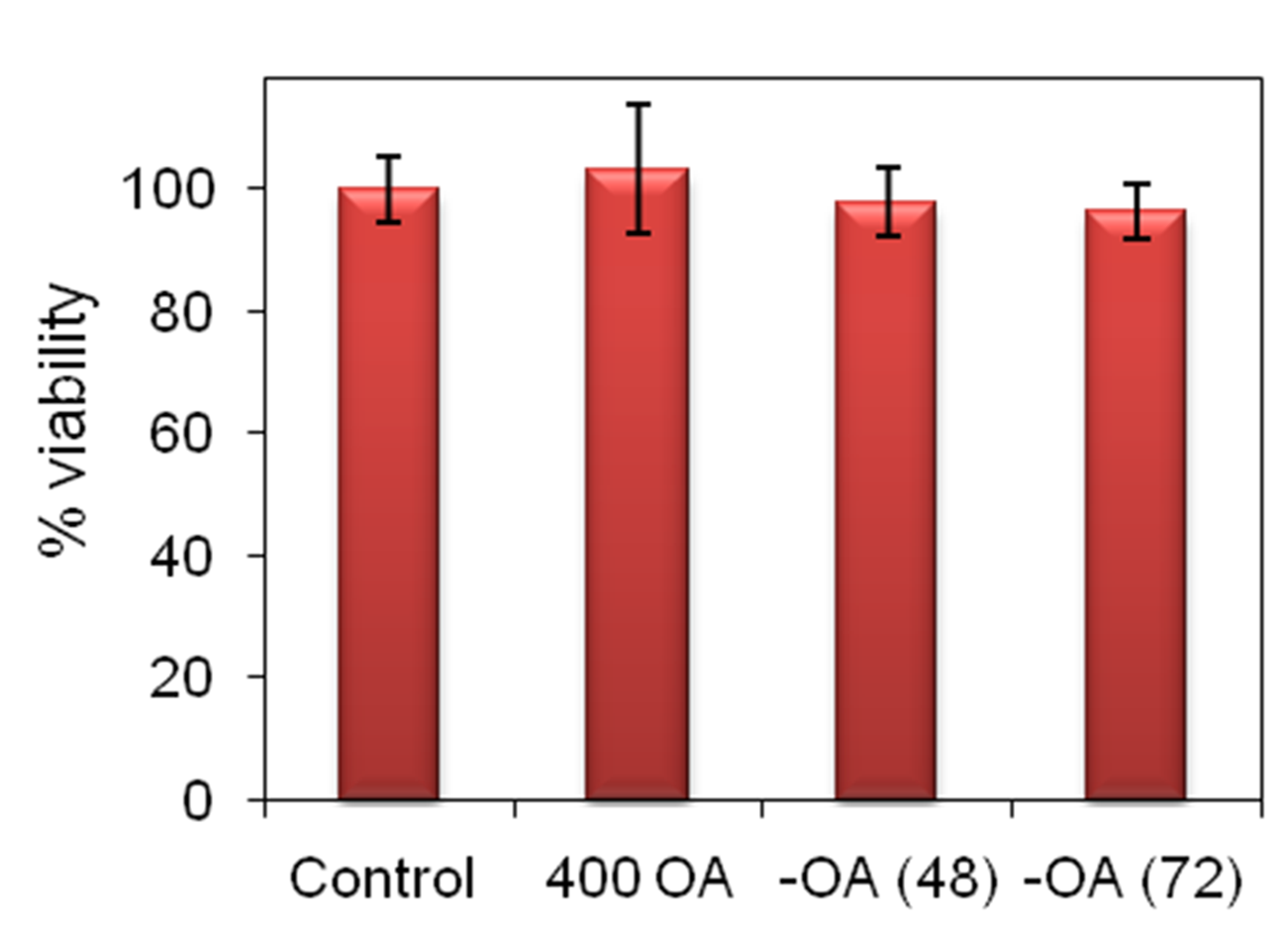

Supplement: S5 Fig — Cell viability was determined by cell counting. The data are expressed as the means ± SD of three independent experiments. The statistical significance of differences among the data was evaluated by ANOVA with post-hoc comparisons of the means via Tukey's honestly-significant-difference test (p <0.05). Key to experimental groups: Control, cells incubated under control culture conditions; 400 OA. cells treated with 400 μM OA for 24 h;–OA (48) and–OA (72), cells treated with 400 μM OA for 24 h and then incubated in the absence of OA for 48 h and 72 h, respectively. In the figure, the percent viability is plotted on the ordinate for the experimental groups indicated on the abscissa. (TIF) [file pone.0170608.s005.tif]

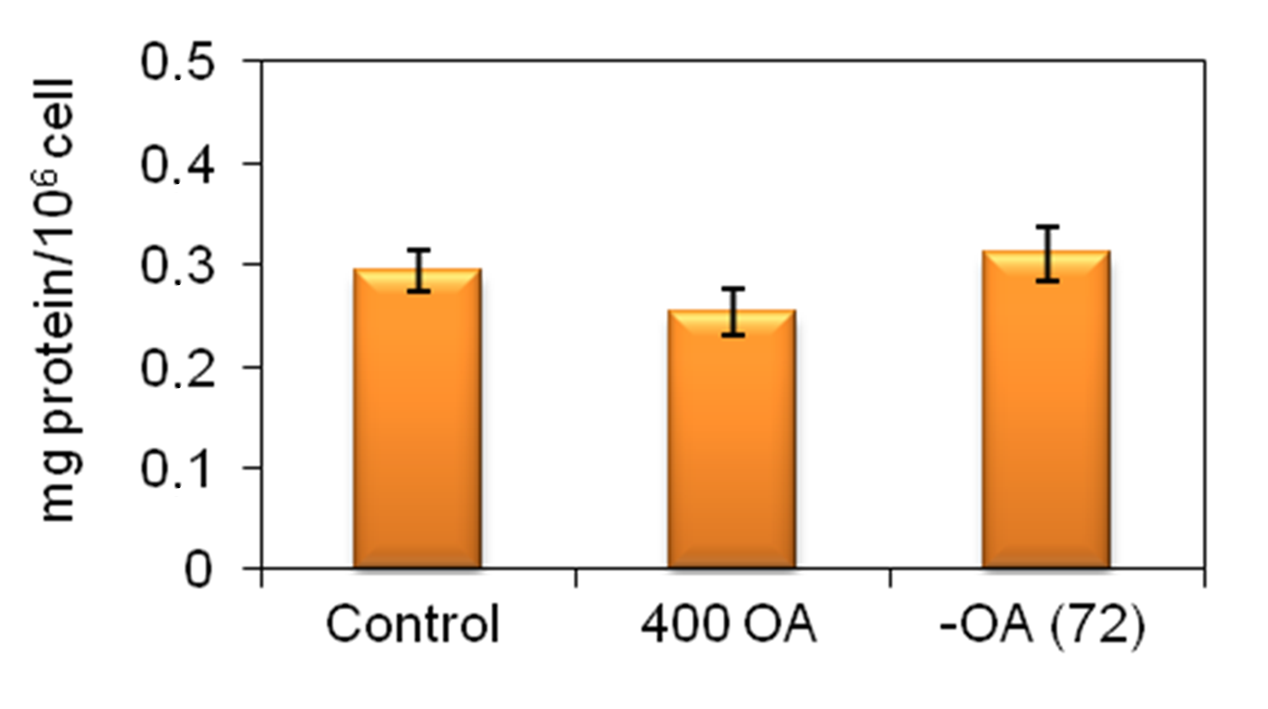

Supplement: S6 Fig — The protein content, expressed as mg of protein per 106 cells, and cell number were determined under the different culture conditions. The data are the means ± SD for three independent experiments in mg protein per 106 cells. The statistical significance of differences among the data was evaluated by ANOVA with post-hoc comparisons of the means via Tukey's honestly-significant-difference test (p <0.05). Key to experimental groups: Control, cells cultured under control conditions; 400 OA, cells treated with 400 μM OA for 24 h; and–OA (72), cells treated with 400 μM OA for 24 h and then incubated in absence of OA for 72 h. In the figure, the cellular-protein content in mg is plotted on the ordinate for the experimental groups indicated on the abscissa. (TIF) [file pone.0170608.s006.tif]
