# Supplementary material for: Reversible Nuclear-Lipid-Droplet Morphology Induced by Oleic Acid: A Link to Cellular-Lipid Metabolism
Source: PLoS One. 2017 Jan 26;12(1):e0170608. doi: 10.1371/journal.pone.0170608 (PMC5268491; doi:10.1371/journal.pone.0170608)
Supplement: S1 Table — The data corresponds to statistical analysis of the cLD size distribution in hepatocytes (Fig 4). Each experimental treatment defined in Fig 4 was compared with the corresponding control condition for the same LD-size category (small, medium, or large; *p<0.05, **p<0.01, ***p<0.001). (DOC) [file pone.0170608.s007.doc]

**S1** Table

| **Statistical analysis of the relative abundance - cLD of hepatocytes** | | | | | | | | |  |
| --- | --- | --- | --- | --- | --- | --- | --- | --- | --- |
| cLD size categories (µm) | | Treatments (%) | | | | | | | |
|  |  | Control | OA 400 | OA 400 + TC 1 | OA 400 + TC 2,5 | OA 400 + TC 5 | -OA (48) | -OA (72) | |
| **S** : | ≤ 0,51 | 56,4 | 4,2*** | 4,9*** | 15,0*** | 52,0 | 51,2 | 54,3 | |
| **M** : | 0,51< y ≤0,77 | 27,4 | 17,1*** | 13,3*** | 27,1* | 30,3 | 25,9 | 31,3 | |
| **L** : | > 0,77 | 16,2 | 78,8*** | 81,8*** | 57,9*** | 17,7 | 22,9* | 14,4 | |

The data corresponds to statistical analysis of the cLD size distribution in hepatocytes (Fig. 2). Each experimental treatment defined in Fig. 2 was compared with the corresponding control condition for the same LD-size category (small, medium, or large; *p<0.05, **p<0.01, ***p<0.001).
