# Supplementary material for: Reversible Nuclear-Lipid-Droplet Morphology Induced by Oleic Acid: A Link to Cellular-Lipid Metabolism
Source: PLoS One. 2017 Jan 26;12(1):e0170608. doi: 10.1371/journal.pone.0170608 (PMC5268491; doi:10.1371/journal.pone.0170608)
Supplement: S2 Table — The data corresponds to statistical analysis of the nLD size distribution in hepatocytes (Fig 4). Each experimental treatment defined in Fig 4 was compared with the corresponding control condition for the same LD-size category (small, medium, or large; *p<0.05, **p<0.01, ***p<0.001). (DOC) [file pone.0170608.s008.doc]

| **S2 Table**  **Statistical analysis of the relative abundance - nLD of hepatocytes** | | | | | | | | |  |
| --- | --- | --- | --- | --- | --- | --- | --- | --- | --- |
| nLD size categories (µm) | | Tratamientos (%) | | | | | | | |
|  |  | Control | OA 400 | OA 400 + TC 1 | OA 400 + TC 2,5 | OA 400 + TC 5 | -OA (48) | -OA (72) | |
| **S** : | ≤ 0,51 | 54,2 | 2,4*** | 14,3 | 40,0 | 53,3 | 54,7 | 64,7 | |
| **M** : | 0,51< y ≤0,77 | 41,7 | 21,4 | 14,3 | 40,0 | 40,0 | 38,8 | 17,6 | |
| **L** : | > 0,77 | 4,2 | 76,2*** | 71,4*** | 20,0 | 6,7 | 6,5 | 17,6 | |

The data corresponds to statistical analysis of the nLD size distribution in hepatocytes (Fig. 2). Each experimental treatment defined in Fig. 2 was compared with the corresponding control condition for the same LD-size category (small, medium, or large; *p<0.05, **p<0.01, ***p<0.001).
