# Supplementary material for: Reversible Nuclear-Lipid-Droplet Morphology Induced by Oleic Acid: A Link to Cellular-Lipid Metabolism
Source: PLoS One. 2017 Jan 26;12(1):e0170608. doi: 10.1371/journal.pone.0170608 (PMC5268491; doi:10.1371/journal.pone.0170608)
Supplement: S3 Table — The data corresponds to statistical analysis of the cLD size distribution in HepG2 cells (Fig 6). Each experimental treatment defined in Fig 6 was compared with the corresponding control condition for the same LD-size category (small, medium, or large; *p<0.05, **p<0.01, ***p<0.001). (DOC) [file pone.0170608.s009.doc]

| **S3 Table**  **Statistical analysis of the relative abundance - cLD of HepG2 cells** | | | | | | | |
| --- | --- | --- | --- | --- | --- | --- | --- |
| cLD size categories (µm) | | Treatments (%) | | | | | |
|  |  | Control | OA 100 | OA 400 | OA 400 + TC 5 | -OA (48) | -OA (72) |
| **S** : | ≤ 0,51 | 30,4 | 15,3*** | 1,7*** | 39,0 | 21,6*** | 53,1*** |
| **M** : | 0,51< y ≤ 0,77 | 50,3 | 22,5*** | 6*** | 36,7*** | 18,7*** | 24,4*** |
| **L** : | >0,77 | 19,3 | 62,2*** | 92,3*** | 24,3 | 59,7*** | 22,5 |

The data corresponds to statistical analysis of the cLD size distribution in HepG2 cells (Fig. 3). Each experimental treatment defined in Fig. 3 was compared with the corresponding control condition for the same LD-size category (small, medium, or large; *p<0.05, **p<0.01, ***p<0.001).
